# The mediating role of self-efficacy in the relationship between past professional training and burnout resilience in medical education: a multicentre cross-sectional study

**DOI:** 10.1186/s12909-024-05854-9

**Published:** 2024-08-14

**Authors:** Rebecca Erschens, Carla Schröpel, Anne Herrmann-Werner, Florian Junne, Lena Listunova, Andrea Heinzmann, Oliver Keis, Katrin Schüttpelz-Brauns, Sabine C. Herpertz, Kevin Kunz, Stephan Zipfel, Teresa Festl-Wietek

**Affiliations:** 1 Department of Psychosomatic Medicine and Psychotherapy, University Hospital Tübingen, Tübingen, Germany; 2 Tübingen Institute for Medical Education, University of Tübingen, Tübingen, Germany; 3 Department of Psychosomatic Medicine and Psychotherapy, Otto von Guericke University Magdeburg, University Hospital Magdeburg, Leipziger Straße 44, 39120 Magdeburg, Germany; 4 Medical Faculty Heidelberg, Office of Students’ Affairs, Heidelberg University, Heidelberg, Germany; 5 Medical Faculty, Albert-Ludwigs-University Freiburg, Office of the Dean of Studies, Freiburg, Germany; 6 Office of the Dean of Studies, Medical Faculty, Ulm University, Ulm, Germany; 7 Medical Education Research Department, Division for Study and Teaching Development, Medical Faculty Mannheim at Heidelberg University, Mannheim, Germany; 8 Department of General Psychiatry, Centre for Psychosocial Medicine, University of Heidelberg, Heidelberg, Germany; 9 Office of Students’ Affairs, University’s Faculty of Medicine, Tübingen, Germany

**Keywords:** Burnout, Self-efficacy, Medical student, Vocational training, Professional experience

## Abstract

**Background:**

Meta-analyses indicate a high prevalence of burnout among medical students. Although studies have investigated different coping strategies and health interventions to prevent burnout, professional experience’s influence on burnout resilience as seldom been explored. Therefore, in our study we aimed to examine the self-efficacy’s mediating role in the relationship between past vocational training and burnout resilience. In the process, we also analysed the associations between study-related variables and burnout resilience.

**Methods:**

In our cross-sectional study, we analysed the data of 2217 medical students at different stages of their university education (i.e. 1st, 3rd, 6th, 10th semester, and final year) at five medical faculties in Germany. The questionnaire included items addressing variables related to medical school, previous professional and academic qualifications, and validated instruments for measuring burnout and self-efficacy.

**Results:**

The overall prevalence of burnout was 19.7%, as defined by high scores for emotional exhaustion and notable values in at least one of the other two dimensions (cynicism or academic efficacy). Higher levels for self-efficacy (*p* < .001), having children (*p* = .004), and financing education with personal earnings (*p* = .03) were positively associated with burnout resilience, whereas having education financed by a partner or spouse (*p* = .04) had a negative association. In a mediation analysis, self-efficacy exerted a suppressor effect on the relationship between vocational training and burnout resilience (indirect effect = 0.11, 95% *CI* [0.04, 0.19]).

**Conclusions:**

Self-efficacy’s suppressor effect suggests that the positive association between vocational training and burnout resilience identified in the mediation analysis disappears for students who have completed vocational training but do not feel efficacious. Those and other findings provide important insights into the psychological mechanisms underlying the development of burnout resilience in medical students and suggest the promotion of self-efficacy in medical education.

## Introduction

The international literature on stress indicates that medical students are vulnerable to a range of psychological complaints. Recent meta-analyses additionally suggest that the prevalence of one such complaint—burnout—among medical students varies considerably, depending on factors such as the instrument used to measure it, its operationalisation, the stage of university education, the country of study and multiple other factors [1–4]. In the context of medical school, stress is moderated by personal and training-related stressors as well as contextual curriculum-related factors. It can be managed or reduced via functional coping strategies, social support, mentoring, and specific health promotion interventions and tends to be perpetuated by dysfunctional behaviour [5–8]. An additional part of the discourse on stress is the recognition that challenges in professional identity formation may significantly contribute to stress among medical students [9–11].

Whether stress peaks during certain semesters or phases of study remains unclear. Some indications suggest a particularly high vulnerability to stress at the beginning at the end and in the transitional stages from preclinical to clinical study [4, 12, 13]. High school students and newly enrolled medical students interested in studying medicine are also subject to such stress [13–15].

Research has suggested that individual personality traits, including an excessive motivation to achieve and be recognised, low self-esteem, a pronounced willingness to overexert oneself, and neuroticism can somewhat predict stress as well as resilience [16–19]. Personality-related factors are indeed crucial in determining an individual’s ability to cope with stress during medical school.

In the context of medical study, investigating coping skills, particularly self-efficacy, is a worthwhile undertaking [20]. To that end, Albert Bandura’s theory of self-efficacy [21, 22] provides a useful framework for understanding the beliefs of medical students. The theory suggests that an individual’s belief in their ability to perform certain actions affects their motivation and capacity to overcome challenges. Students with higher self-efficacy may be better equipped to handle the demanding nature of medical school and therefore be more resilient against burnout [21, 22]. The international literature on medical students and other students in the health sciences provides clear evidence supporting self-efficacy’s role as a protective factor against burnout. Moreover, numerous studies have demonstrated that higher self-efficacy is associated with a lower susceptibility to burnout, in relationships that are consistent across cultural contexts and national education systems. According to the literature, students in medicine and the health sciences with a high level of self-efficacy demonstrate a heightened ability to cope with the demands of both study and clinical practice [20, 23, 24].

Analysing the role of different portfolios of competencies in combination with self-efficacy can afford profound insights into the different coping mechanisms of medical students. One example is academic and professional experience, which can characterise students in highly individual ways and influence their thoughts and actions during their studies as well as later in their professional lives [25, 26].

In Germany, professional experience can be an advantage for admission to medical studies. Such experiences include vocational training and work experience in the medical field, volunteer work, participation in competitions and awards earned, results of validated aptitude tests, results of situational judgement tests, and even interviews [25, 27]. However, research on the relationship between professional and academic qualifications earned and examination- and grade-based academic success in medical studies has shown heterogeneous results [26]. Nevertheless, the overall results seem to indicate that pre-existing qualifications do not provide an edge in academic success [25, 28, 29].

That ambiguity opens up the possibility of deepening the investigation into professional experience at a psychological level and illuminating the individual coping strategies of medical students. Per Bandura’s theory [21, 22], an individual’s beliefs about their ability to cope with tasks significantly impact their actions and emotional well-being. Thus, pre-existing professional qualifications could serve as a starting point for examining the extent to which they influence the individual coping strategies of future medical students. A psychological perspective should also be integrated to gain a deeper understanding of the complex dynamics behind the apparent lack of any clear link between past professional qualifications and traditional parameters of medical study. Thus, in our study, we aimed to identify potential implications for promoting psychological resilience and self-efficacy among medical students.

The aim of our study was to investigate the relationship between preexisting professional and academic qualifications, self-efficacy, socio-demographic and medical school-related variables with resilience against burnout, or ‘burnout resilience’, among students at different stages of their university career. In particular, we wanted to answer the following research question:

(i) How are different socio-demographic variables, medical school-related variables, professional and academic pre-qualifications, and self-efficacy associated with burnout resilience?

According to Bandura’s theory of self-efficacy [21, 22], past professional training can provide a wealth of experience that strengthens an individual’s belief in their ability to cope with the demands of medical school and everyday clinical practice. Bandura’s theory therefore supports the assumption that medical students who have completed a vocational training program may have increased self-efficacy, because subject knowledge may positively influence students’ perceived self-efficacy [30]. The theory also emphasises the contextual transfer of self-efficacy. Students who have previously strengthened their self-efficacy in professional contexts could transfer those beliefs to medical school, which may help them to perceive their abilities as being equally effective for their studies and, in turn, could reduce their vulnerability to burnout.

Erikson’s theory of identity development [31] complements those considerations by examining psychodynamic processes in the context of self-discovery and emphasising the significance of identity formation during adolescence and beyond. The theory includes several stages, with the period between 20 and 40 years of age being a crucial stage for further identity development. Medical students with preexisting vocational qualifications may have already developed a more stable professional identity before studying medicine. In turn, that development could strengthen their self-image and support their orientation towards a professional future, which could consequently promote their burnout resilience. From a psychodynamic perspective, self-efficacy is not only viewed as a cognitive belief but also as an emotional foundation with deep roots in individual experiences. Recognising and incorporating past achievements in vocational training can enhance self-esteem and thus strengthen self-efficacy in medical school.

Schwarzer’s [32] consideration of psychological stress management highlights the importance of self-efficacy in coping with psychological stress. Medical students who perceive themselves as being more efficacious may be better equipped to handle the demands of their studies and have greater resilience against stress-related pressures. Therefore, we also sought to answer a second research question, as follows:

(ii) Does self-efficacy mediate the relationship between past vocational training and burnout resilience?

## Materials and methods

### Sample and procedure

In our study, we conducted an online survey of medical students at five medical schools in Germany. The survey was part of a larger research project within the framework of ‘Studying Successfully in Baden-Württemberg – Funding Line 4: Aptitude and Selection’. Further research into the relationship between academic and professional pre-qualifications and the academic success of medical students from the third semester onwards has been conducted as part of the mentioned project and been published elsewhere [25]; thus, similarities may exist between the description of the study procedure and the descriptions of the sample. Three groups of participants were involved: (i) preclinical medical students (i.e. in their first and third semesters), (ii) clinical medical students (i.e. in their sixth and tenth semesters), and (iii) medical students in their final year. The survey was conducted anonymously online using EvaSys and not administered during high-stress exam periods.

### Measures

The questionnaire comprised questions to collect socio-demographic data, including age, gender, marital status, and children, as well as questions about medical school, including current semester level, means of financing of medical study (multiple responses possible), undergraduate grade point average (GPA), and the country where the undergraduate GPA was earned. Participants were asked if they had completed vocational training (i.e. with final grades available) in the medical field before medical school or had other pre-qualifications, including volunteer work and/or any academic degrees.

#### Professional and academic pre-qualifications

Medical students answered questions about any practical experience that they had prior to medical school. They were also asked about any vocational training or volunteer work that they had completed and, if present, then about the specific field(s) in which they had gained experience. In Germany, depending on the medical school, work experience, volunteer work, and vocational training completed in the medical field may be advantageous for admission [25, 27]. We also inquired about whether they had completed a degree in another subject before commencing medical school. On that count, we established three dichotomous variables based on the following definitions: *vocational training* was defined as professional training completed (i.e. with a grade) in the medical field, an *academic degree* was defined as a completed bachelor’s or master’s degree with a grade, and *volunteer work* was defined as having worked voluntarily for at least 11 consecutive months.

#### Student burnout

The Maslach Burnout Inventory Student Survey (MBI-SS), developed by Schaufeli et al. [33] for students and convert into a German version by Gumz et al. [34], comprises 15 items on three scales: Emotional Exhaustion (EE, 5 items), Cynicism (CY, 4 items), and Academic Efficacy (AE, 6 items). The items are rated on a 7-point Likert scale ranging from 0 (*never*) to 6 (*daily*), and Cronbach’s alphas for the scales ranges from α = 0.81 to α = 0.86 [34].

To operationalise *burnout* according to a more conservative definition (see [5, 41]), we created a dichotomous variable such that burnout indicated having a high score for EE (i.e. score ≥ 16) and having notable values in at least one of the other two dimensions (i.e. CY score ≥ 10 or AE score ≤ 23).

#### Burnout resilience

In accordance with our definition of burnout, we defined burnout resilience as a complementary variable, which resulted in a dichotomous variable comprising the values burnout (0) and burnout resilience (1). It is essential to acknowledge that we do not consider burnout resilience to be a long-term concept, such as burnout recovery.

#### General self-efficacy

The General Self-Efficacy Scale (GSE) [35] is a validated inventory that measures an individual’s subjective beliefs and expectations regarding their ability to cope with challenging situations based on their own competencies. The GSE comprises 10 concise statements (e.g. ‘I can find a solution to any problem’ and ‘I always know how to act in unexpected situations’) with responses ranging from 1 (not at all true) to 4 (exactly true) on a 4-point Likert scale. The GSE produces a total score between 10 and 40 points, with higher scores indicating higher self-efficacy, and has shown high reliability, validity, and internal consistency [36, 37]. The Cronbach’s alpha of the GSE ranges from 0.76 to 0.90 [36, 37].

### Statistical analysis

#### General and descriptive statistics

We used SPSS version 28.0.0.0 (IBM, Armonk, NY, USA) for statistical analyses with significance set at α < 0.05. For descriptive analysis, means and frequencies were calculated for all socio-demographic variables, professional and academic qualifications, self-efficacy, burnout and burnout resilience, and for all individual scales of the MBI-SS. The variable of cynicism was found to significantly violate normal distribution, as revealed by graphical tests using histograms; therefore, medians were calculated along with means. Within the operationalisation of burnout and resilience to burnout, all MBI scales were included, but not as a metric sum score, but as a dichotomous variable (see description above). Other studies [5, 41] have adopted or discussed a similar operationalisation and analysis strategy.

In addition, we analysed whether there were differences between semester levels on all scales of the MBI, burnout and burnout resilience, and self-efficacy. To identify differences between two groups, we conducted χ^2^-tests or independent *t*-tests; by contrast, to identify differences between more than two groups, we conducted a one-way ANOVA or χ^2^-tests. When homogeneity of variances could not be assumed using Levene’s test, we calculated Welch’s test or Welch’s ANOVA. We interpreted all effect sizes according to Cohen’s [38] guidelines. The effect sizes of η^2^ = 0.01, Cramér’s V = 0.1 and |*d*| = 0.2 indicate a small effect. Similarly, effect sizes of η^2^ = 0.06, V = 0.3 and |*d*| = 0.5 indicate a medium effect, while effect sizes of η^2^ = 0.14, V = 0.5 and |*d*| = 0.8 indicate a large effect.

#### Logistic regression analysis

**Fig. 1:**
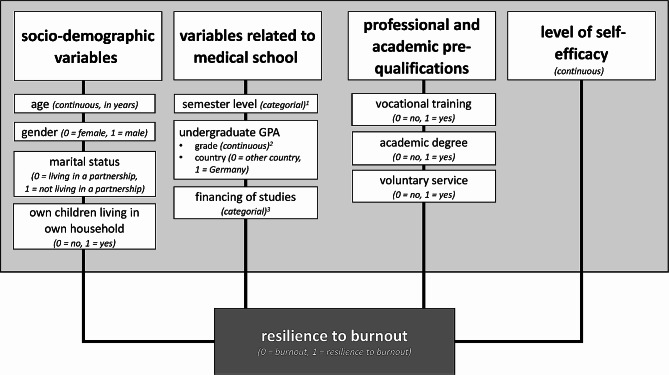
Independent variables (i.e. continuous and categorial) used in the regression analysis to predict burnout resilience^1^ categories: 1rd semester, 3rd semester, 6th semester, 10th semester, final year. ^2^lower values indicate better grades. ^3^categories: multiple choice, financing studies by parents/relatives (0 = no, 1 = yes), financing studies by partner/spouse (0 = no, 1 = yes), financing studies by own earnings (0 = no, 1 = yes), financing studies by savings (0 = no, 1 = yes)

We conducted a logistic regression analysis to determine the impact of various socio-demographic variables, variables related to medical school, professional and academic pre-qualifications, and self-efficacy on burnout resilience. Figure 1 provides details about the variables analysed and their coding. The first category was chosen as the reference category for the categorical variables. Odds ratios (*OR*) were calculated to determine effect sizes; an *OR* of approximately 1.5 indicates a small effect, an *OR* of approximately 3.0 indicates a medium effect, and an *OR* of approximately 5.0 indicates a large effect. If any *OR *exceeds 1, then the value has to be inverted (1/*OR*) [39].

#### Mediation analysis

We conducted a mediation analysis using the PROCESS Macro by Hayes [40] to determine whether self-efficacy mediates the relationship between completing vocational training in the medical field before medical school and burnout resilience among medical students. Because the outcome variable was dichotomous, effect sizes could not be calculated. However, prior to conducting the mediation analysis, we also performed exploratory analyses by calculating descriptive statistics, Welch *t*-tests, and χ^2^-tests to examine the associations and effects among the three variables. The results of univariate analysis were used to better understand the effects of the mediation analysis.

## Results

### Response rate and sample description

The final calculations were based on a sample size of 2217 medical students, with a response rate of 48.4%. A total of *n* = 1422 women (64.7%), *n* = 772 men (35.1%), and *n* = 4 students (0.2%) who indicated ‘other’ for gender participated in the study. The mean age was 23.89 years (*SD* = 3.88) and ranged from 16 to 45 years. Overall *n* = 316 medical students (14.3%) were in their 1st semester, *n* = 432 (19.5%) were in their 3rd semester, *n* = 619 (27.9%) were in their 6th semester, *n* = 394 (17.8%) were in their 10th semester, and *n* = 456 (20.6%) were in their final year.

Overall, *n* = 531 students (24.0%) reported having completed vocational training in the medical field, with the majority being paramedics (*n* = 230, 43.3%), followed by nurses (*n* = 194, 36.5%). Meanwhile, *n* = 133 students (6.0%) reported having completed an academic degree prior to starting medical school; most of those participants held a degree in a STEM - (abbr. for Science, Technology, Engineering and Maths) subject (*n* = 46, 34.6%), followed by the medical field (e.g. dentistry, molecular medicine, and human biology; *n* = 33, 24.8%) and psychology (*n* = 18, 13.5%). Beyond that, *n* = 468 participants (21.2%) reported completing volunteer work prior to medical school, most often in healthcare (*n* = 182, 39%), followed by child and youth welfare (*n* = 48, 10.3%) and care for people with disability (*n* = 23, 4.9%).

### Burnout among students at different semester levels

Using a rather conservative definition of *burnout*, (for an overview see Dyrbye and Shanafelt [41]), we calculated a burnout rate of 19.7% (*n* = 429) compared with an 80.3% (*n* = 1751) rate of burnout resilience. The highest percentage *for burnout* surfaced among medical students in their 3rd semester (22.3%) and the lowest percentage among students in their 10th semester (13.9%). Moreover, 31.0% of students reported scores of emotional exhaustion ≥ 16, with the highest percentage in the 3rd (42.5%) and 1st semester (41.2%) and the lowest percentage in the 10th semester (21.3%). Additionally, 22.7% of all students reported cynicism scores ≥ 10, with the highest percentage in the final year (29.6%) and the lowest percentage in the 1st semester (14.7%). Overall, 31.4% of the students reported low academic efficacy scores with ≤ 23, with the highest percentage in the 6th semester (35.8%) and the lowest percentage in the 1st semester (24.3%).

### Results of self-efficacy

The mean sum score for self-efficacy was 30.96 (*SD* = 4.04) with a range from *M* = 30.47 (*SD* = 4.48) for medical students in final year and *M* = 31.23 (*SD* = 4.08) for medical students in their 10. Semester. Self-efficacy levels significantly differed between semesters, *p* = .02 with η^2^ = 0.005, which is below the threshold for a small effect size.

Table 1 displays the means, frequencies, and additional descriptive statistics of the Maslach Burnout Inventory and self-efficacy scores for students grouped by current semester level.

**Table 1:** Descriptive statistics of the Maslach Burnout Inventory and self-efficacy scores for the different semester groups

		total sample	1st semester	3rd semester	6th semester	10th semester	final year	*p*
emotional exhaustion (EE)								
	$\:{M}_{sum}$(*SD*)^1^	12.45(6.26)	14.34(6.07)	14.35(6.02)	12.40(6.15)	10.51(5.85)	11.09(6.24)	< 0.001***, η^2^ = 0.01
	$\:{M}_{scal}$(*SD*)^2^	2.49(1.25)	2.87(1.21)	2.87(1.20)	2.48(1.23)	2.10(1.17)	2.22(1.25)	
	EE ≥ 16; *n*(%)	675(31.0%)	127(41.2%)	181(42.5%)	179(29.5%)	82(21.3%)	106(23.6%)	< 0.001***, *V* = 0.17
cynicism (CY)								
	$\:{M}_{sum}$(*SD*)	5.88(5.34)	4.32(4.86)	5.09(4.70)	6.14(5.42)	6.30(5.36)	6.99(5.76)	< 0.001***, η^2^ = 0.03
	*Mdn* ^*5*^	4	3	4	5	5	6	
	$\:{M}_{scal}$(*SD*)	1.47(1.34)	1.08(1.21)	1.27(1.18)	1.53(1.35)	1.58(1.34)	1.75(1.44)	
	CY ≥ 10; *n*(%)	495(22.7%)	45(14.7%)	67(15.8%)	154(25.4%)	96(24.6%)	133(29.6%)	< 0.001***, *V* = 0.11
academic efficacy (AE)								
	$\:{M}_{sum}$(*SD*)	25.80(5.43)	26.91(5.24)	26.15(5.43)	25.05(5.30)	26.01(5.33)	25.56(5.64)	< 0.001***, η^2^ = 0.01
	$\:{M}_{scal}$(*SD*)	4.30(0.90)	4.49(0.87)	4.36(0.90)	4.17(0.88)	4.33(0.89)	4.26(0.94)	
	AE ≤ 23; *n*(%)	671(31.4%)	71(24.3%)	118(28.0%)	215(35.8%)	118(30.9%)	149(33.6%)	0.004**, *V* = 0.09
burnout^3^								0.02*, *V* = 0.07
burnout	*n*(%)	429(19.7%)	63(20.9%)	95(22.3%)	133(21.7%)	54(13.9%)	84(18.7%)	
resilience to burnout^4^	*n*(%)	1751(80.3%)	239(79.1%)	331(77.7%)	481(78.3%)	334(86.1%)	366(81.3%)	
self-efficacy^5^	*M*(*SD*)	30.96(4.04)	30.76(3.56)	31.10(3.78)	31.16(4.06)	31.23(4.08)	30.47(4.48)	0.02*, η^2^ = 0.005

^1^$\:{M}_{sum}$(*SD*) describes the mean value (with standard deviation) of the respective sum score of EE (range 0–30), CY (range 0–24), AE (range 0–36); ^2^$\:{M}_{scal}$(*SD*) describes the mean value of the scale score EE (range 0–5), CY (range 0–4), AE (range 0–6); ^3^burnout was defined with high EE (score ≥ 16) plus high CY (score ≥ 10) or low AE(score ≤ 23); ^4^resilience to burnout was defined as a complementary expression of burnout, ^5^sum score range self-efficacy scale: between 10 and 40; *p < .05, *p* < .01, *** *p* < .001

### Results of logistic regression analysis for the sample

All assumptions for conducting a logistic regression analysis were met. The continuous variables exhibited a linear relationship with the dependent variable, and no outliers (i.e. studentised residuals ± 3 *SD*) or multicollinearity (i.e. *r* < .70) emerged between the independent variables. The analysis included 2002 participants from our sample, with 215 students excluded due to missing values. A logistic regression analysis was conducted to investigate the impact of various socio-demographic variables (i.e. age, gender, marital status, and having children), variables related to medical school (i.e. semester, undergraduate GPA, country of graduation, and financing of medical study), professional and academic pre-qualifications (i.e. vocational training, academic degree, and volunteer work), and self-efficacy on burnout resilience. The statistical model was significant, χ^2^(18) = 200.10, *p* < .001, with a small effect size (*f*^2^ = 0.18) per Cohen’s [38] guidelines.

To answer our first research question, we found a significant positive association of resilience to burnout with having own children living in the same household, with financing studies with own income, and with higher levels of self-efficacy. The odds of reporting burnout resilience were higher for students who had their own children living in the same household (*p* = .004, *OR* = 4.26) and for students who were financing their medical study with their own earnings (*p* = .03, *OR* = 1.34). Additionally, higher levels of self-efficacy were associated with a greater chance of reporting burnout resilience (*p* < .001, *OR* = 1.20). We found a significant negative association between burnout resilience and financing studies with the help of a partner or spouse. Having a partner or spouse finance medical study was found to decrease the chance of reporting burnout resilience (*p* = .04, *OR* = 0.55, 1/*OR* = 1.82). However, there was no association with age, gender, marital status, semester level, undergraduate GPA, country of graduation, financing studies by parents/relatives, financing studies by savings, vocational training in the medical field, an academic degree or voluntary service.

Table 2a presents all coefficients and *OR*s, while Table 2b presents the means or frequencies of all variables analysed in the regression analysis.

**Table 2a:** Results of the logistic regression analysis predicting resilience to burnout

					95%CI(OR)	
	B	SE	*p*	OR	LL	UL
predictors						
(intercept)	-4.27	0.82	< 0.001	0.01		
age	< 0.01	0.03	0.95	1.00	0.94	1.06
gender	0.01	0.13	0.91	1.01	0.78	1.32
marital status	0.01	0.13	0.91	1.01	0.79	1.31
own children (living in own household)	1.45	0.51	0.004	4.26	1.58	11.49
1st semester (reference)						
3rd semester	-0.13	0.21	0.52	0.88	0.59	1.31
6th semester	-0.14	0.22	0.53	0.87	0.57	1.33
10th semester	0.38	0.27	0.16	1.46	0.86	2.47
final year	0.24	0.28	0.38	1.27	0.74	2.18
undergraduate GPA	-0.13	0.17	0.44	0.87	0.62	1.23
country of graduation	0.10	0.24	0.68	1.10	0.69	1.76
financing studies by parents/relatives	0.30	0.18	0.11	1.34	0.94	1.93
financing studies by partner/spouse	-0.60	0.30	0.04	0.55	0.31	0.98
financing studies by own earnings	0.29	0.13	0.03	1.34	1.03	1.74
financing studies by savings	0.08	0.14	0.55	1.08	0.83	1.41
vocational training in the medical field	-0.20	0.19	0.29	0.82	0.56	1.19
academic degree	-0.24	0.28	0.38	0.78	0.45	1.35
voluntary service	-0.28	0.15	0.05	0.75	0.57	1.00
self-efficacy	0.18	0.02	< 0.001	1.20	1.16	1.24

Note. The binary outcome variable was resilience to burnout (0 = burnout, 1 = resilience to burnout)

**Table 2b:** Means and frequencies separately for individuals reporting resilience to burnout versus burnout

variables	burnout	resilience to burnout
age	*M* = 23.89 (*SD* = 3.87)	*M* = 23.83 (*SD* = 3.82)
gender		
female	*n* = 271(20.9%)	*n* = 1025(79.1%)
male	*n* = 123(17.4%)	*n* = 583(82.6%)
marital status		
partnership	*n* = 186(19.5%)	*n* = 770(80.5%)
no partnership	*n* = 208(19.9%)	*n* = 838(80.1%)
own children (living in own household)		
no	*n* = 388(20.0%)	*n* = 1552(80.0%)
yes	*n* = 6(9.7%)	*n* = 56(90.3%)
1st semester		
no	*n* = 335(19.5%)	*n* = 1386(80.5%)
yes	*n* = 59(21.0%)	*n* = 222(79.0%)
3rd semester		
no	*n* = 308(19.0%)	*n* = 1314(81.0%)
yes	*n* = 86(22.6%)	*n* = 294(77.4%)
6th semester		
no	*n* = 271(18.8%)	*n* = 1169(81.2%)
yes	*n* = 123(21.9%)	*n* = 439(78.1%)
10th semester		
no	*n* = 344(20.9%)	*n* = 1299(79.1%)
yes	*n* = 50(13.9%)	*n* = 309(86.1%)
final year		
no	*n* = 318(20.1%)	*n* = 1264(79.9%)
yes	*n* = 76(18.1%)	*n* = 344(81.9%)
undergraduate GPA	*M* = 1.49 (*SD* = 0.54)	*M* = 1.43 (*SD* = 0.52)
country of graduation		
Germany	366(19.7%)	1489(80.3%)
other country	28(19.0%)	119(81.0%)
financing studies by parents/relatives		
no	*n* = 72(23.7%)	*n* = 232(76.3%)
yes	*n* = 322(19.0%)	*n* = 1376(81.0%)
financing studies by partner/spouse		
no	*n* = 370(19.4%)	*n* = 1536(80.6%)
yes	*n* = 24(25.0%)	*n* = 72(75.0%)
financing studies by own earnings		
no	*n* = 183(23.0%)	*n* = 614(77.0%)
yes	*n* = 211(17.5%)	*n* = 994(82.5%)
financing studies by savings		
no	*n* = 270(19.4%)	*n* = 1119(80.6%)
yes	*n* = 124(20.2%)	*n* = 489(79.8%)
vocational training in the medical field		
no	*n* = 287(18.9%)	*n* = 1231(81.1%)
yes	*n* = 107(22.1%)	*n* = 377(77.9%)
academic degree		
no	*n* = 369(19.6%)	*n* = 1512(80.4%)
yes	*n* = 25(20.7%)	*n* = 96(79.3%)
voluntary service		
no	*n* = 291(18.5%)	*n* = 1284(81.5%)
yes	*n* = 103(24.1%)	*n* = 324(75.9%)
self-efficacy	*M* = 28.72 (*SD* = 4.40)	*M* = 31.55 (*SD* = 3.74)

Note. Burnout was defined with high EE (score ≥ 16) plus high CY (score ≥ 10) or low AE (score ≤ 23) and resilience to burnout was defined complementary

### Mediation analysis

When conducting our mediation analysis, we followed Baron and Kenny’s [42] four steps for establishing mediation. Because the dependent variable was dichotomous, it was impossible to calculate the total effect between vocational training and burnout resilience. However, Rucker et al. [43] have argued that a total effect is not necessary for mediation analysis. Our analysis included 2133 cases (i.e. with 84 missing values). Preliminary univariate analyses showed that, on average, self-efficacy was higher in the group that completed vocational training (*M* = 31.44, *SD* = 3.71) than in the group without vocational training (*M* = 30.84, *SD* = 4.13), Welch’s *t*(954.81) = − 3.12, *p* = .002. However, the effect size was very small (*d* = − 0.15). The group with burnout resilience had a higher average self-efficacy (*M* = 31.55, *SD* = 3.73) than the group with symptoms of burnout (*M* = 28.64, *SD* = 4.41), with a medium effect size (*d* = − 0.75) determined using Welch’s *t* test, *t*(569.10) = − 12.43, *p* < .001. No significant association appeared between burnout resilience and the completion of vocational training, χ^2^(1) = 3.72, *p* = .05. Of all 516 medical students with vocational training, 77.5% (*n* = 400) indicated burnout resilience. Similarly, of all 1617 medical students without vocational training, 81.4% (*n* = 1316) indicated burnout resilience.

**Fig. 2:**
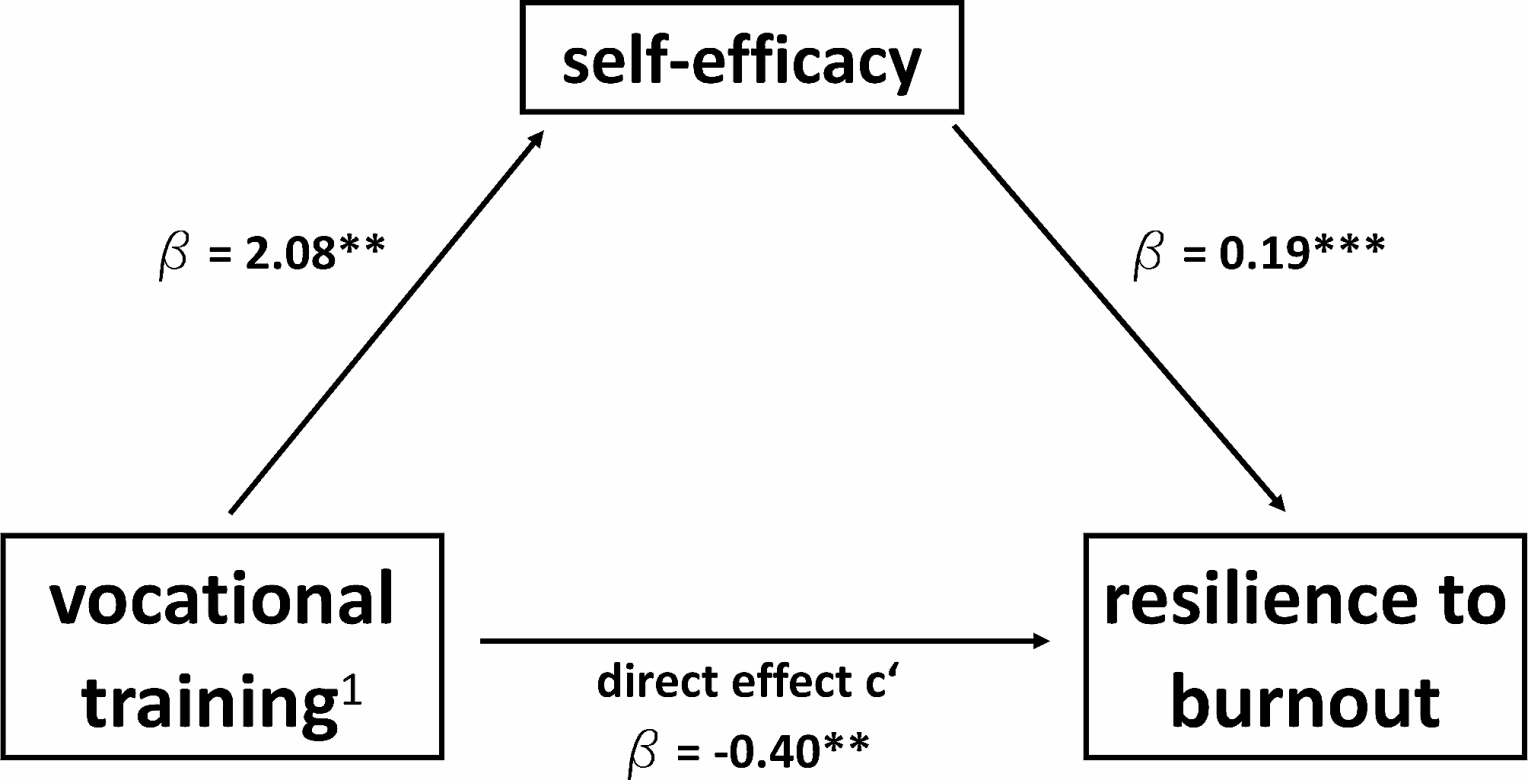
The mediation analysis according to Baron and Kenny’s [42] four steps showed that vocational training (0 = no, 1 = yes) predicted self-efficacy, which in turn predicted resilience to burnout (0 = no, 1 = yes). After including self-efficacy as a mediator variable in the model, the direct effect c’ remained significant^1^ training (in the medical field) was one of three variables that we defined as professional and academic pre-qualifications (see Method section); in the mediation analysis we analysed exclusively the association with prior vocational training ^**^p < .01, ^***^p < .001

To test the assumptions of our logistic regression analysis, we examined the linear relationship between the metric variable of self-efficacy and the dichotomous dependent variable. We found no outliers (i.e. studentised residuals ± 3 *SD*). After including the mediator in the model, vocational training significantly predicted self-efficacy (β = 2.08, *p* = .002), which in turn significantly predicted burnout resilience (β = 0.18, *p* < .001). After the mediator was incorporated into the model, the direct effect c’ remained significant (β = − 0.40, *p* = .002). In line with our second research question, we found that the relationship between vocational training and burnout resilience was mediated by self-efficacy, with an indirect effect of 0.11 and a 95% CI of [0.04, 0.19]. Figure 2 provides an overview of the mediation process.

## Discussion

In our multicentre, cross-sectional study, we investigated how different socio-demographic variables and variables related to medical study, professional and academic qualifications, and self-efficacy relate to burnout resilience and whether self-efficacy mediates the relationship between past vocational training and burnout resilience. In this section, we present our findings and discuss their implications in the context of relevant international literature and theoretical models.

### Burnout prevalence at different stages of university education

We employed a rather conservative definition of *burnout*, which we determined using high scores for emotional exhaustion in addition to notable values in at least one of two other dimensions (high scores for cynicism or low scores for academic efficiency). The overall burnout rate was 19.7%, with rates ranging from 13.9% for students in their 10th semester to 22.3% for students in their 3rd semester. The rate of emotional exhaustion was highest in the early stages and lowest in the 10th semester. The rate of cynicism was highest in the final year of study and lowest in the 1st semester. The 6th semester had the highest frequency of low academic efficiency, while the 1st semester had the lowest.

The study’s findings align with the heterogenous results in national and international literature on burnout among students [1, 2, 44, 45]. Furthermore, the survey of burnout (resilience) across numerous semester groups offers insights into the prevalence and diverse manifestations of this construct throughout the course of the study. According to Lazarus and Folkman’s [46] stress model, our results suggest that emotional exhaustion among students during earlier semesters is due to the initial adjustment to the demands of studying. By contrast, students in later semesters may have already developed coping strategies that enable them to better manage stress. An increasing rate of cynicism in later semesters could indicate a process of disillusionment or negative adjustment to the academic environment. Festinger’s [47] concept of cognitive dissonance may explain that phenomenon. Beyond that, differences in academic efficiency may relate to developmental and maturational processes that influence self-concept and self-regulation, as described in Bandura’s self-efficacy theory [21, 22]. The findings of the present study are in alignment with those reported in the most recent systematic literature review on burnout [2]. Those findings are valuable for developing targeted prevention and intervention strategies to promote students’ well-being and performance during their studies.

### Relationship between burnout resilience with variables related to medical school

Our regression analysis to answer the first research question revealed no significant relationship between burnout resilience and age, gender, marital status, semester, country of graduation, means of financing medical study, vocational training completed in the medical field, university degree, or volunteer work before medical study. However, burnout resilience was positively related to a higher level of self-efficacy, having children of one’s own living in the same household, and financing one’s studies with one’s own income. By contrast, having a partner or spouse finance medical study was negatively associated with burnout resilience.

The results of our regression analysis offer valuable insights into the factors of burnout resilience among medical students. Other studies have also supported the relationship between higher levels of self-efficacy and burnout resilience in medical students, final year medical students and residents. These studies emphasise the significance of fostering self-efficacy in the initial stages of medical training and identifying indications of burnout in medical students. The inclusion of prevention programmes in the curriculum was also recommended in order to minimise the psychological burden on young professionals. [2, 23, 24, 48, 49].

According to Bandura’s self-efficacy theory, confidence in one’s abilities plays a central role in coping with stress and challenges [21, 22]. Moreover, the study suggests a positive correlation between financing one’s own studies and burnout resilience, indicating that financial independence may contribute to a sense of control and stability. Those findings align with Julian Rotter’s control belief theory [50]. Conversely, the negative association between a partner or spouse financing studies and burnout resilience could indicate potential conflicts or dependencies within the partnership. The concept of interpersonal dynamics and stress transfer within relationships can shed light on this issue: Stress or conflict in one partner can affect the mental health and well-being of the other, leading to burnout [51, 52]. Other studies have also demonstrated a correlation between stress and financial difficulties [53, 54].

Concepts and analysis with focus on conflict moderation can explain why completing vocational training, volunteer work, or an academic degree in the medical field prior to studying medicine is not related to burnout resilience. The relationship between different domains of life, including work and education, and factors of mental health such as burnout resilience may moderated by the extent to which those areas conflict with each other and may be particularly relevant to understanding the experience of medical students, who often have high professional expectations and undergo intensive academic training associated with high demands and stress [2, 16, 55–57]. Individuals who have gained professional experience in the medical field may have developed perceptions or standards that do not always align with the requirements of medical school [26]. Conflict and a negative impact on burnout resilience can therefore arise when professional experience and the requirements of a medical curriculum clash. Although there may be no direct link between professional and academic qualifications in the medical field and burnout resilience, the potential conflict described warrants attention. The potential stress resulting from the conflict between professional expectations and the requirements of study may outweigh the potential benefits of preexisting professional experience for the burnout resilience of medical students. It is important to consider that dynamic when evaluating the impact of preexisting professional experience on burnout resilience [58–60].

### Mediation analysis and the suppressor effect

The results of our mediation analysis to answer the second research question suggest that completing professional training in the medical field is associated with higher levels of self-efficacy. Increased self-efficacy was also positively associated with burnout resilience. Our findings suggest that self-efficacy mediates the relationship between vocational training and burnout resilience. They additionally indicate a suppressor effect of the mediator in that relationship given the opposite signs of the direct effect and the mediated effect [61]. Medical students who have undergone professional training in the medical field and who reported higher levels of self-efficacy also exhibited greater burnout resilience. Completing professional training may have increased their burnout resilience. However, the positive relationship between professional training and burnout resilience may disappear or even turn negative if students do not feel efficacious. A higher level of self-efficacy can help students to cope better with the demands of medical school, which may consequently increase their burnout resilience (see also [21–24]). Professional pre-medical training can therefore be viewed as a form of education that provides students with specific skills and competencies. As a result of their training, students may feel better equipped to face the challenges of studying medicine [30]. The conceptualisation of self-efficacy as the result of experiences and the acquisition of skills, as outlined in Bandura’s self-efficacy theory [21], supports that interpretation. However, the suppressor effect suggests that completing training does not automatically lead to higher self-efficacy, for only the combination of vocational training and higher self-efficacy yields burnout resilience. Self-efficacy’s importance as a mediating variable in stress management processes [62] is emphasised by its role in mediating the relationship between vocational training and burnout resilience and suggests that self-efficacy plays a crucial role in mitigating the negative effects of stressors and promoting resilience.

### Strengths and limitations

The present study examined the relationship between self-efficacy, past professional experience and burnout resilience in 2217 undergraduate medical students in their first, third, sixth and tenth semester and in their final year of university education. A response rate of 48.4% was achieved. To the best of our knowledge, no other studies have been conducted that examine these relationships in a multicentre study at five medical faculties in Germany.

One of the study’s limitations was that the associations were analysed following a cross-sectional design. Therefore, the reported associations cannot be interpreted causally but only associatively. In future studies, researchers should use a longitudinal design to validate these relationships. It is also important to note that the students in the study reported their experience of burnout using a valid self-report instrument. No clinical interviews were conducted, nor were any assessments made by external sources.

Given the limited scope of this article, it is not feasible to delve into the comprehensive discourse surrounding the nature, operationalisation and classification of burnout within the context of classification systems such as the ICD-11 or the DSM-V. In addition, in the present study we used a dichotomous variable to define burnout and burnout resilience.

All three of the individual scales were considered in this process. We would like to point out that the MBI was not originally designed to provide an (additive) overall burnout score and that the emotional exhaustion, cynicism and academic efficacy scales should be considered individually [33, 34]. In their systematic review, Erschens and colleagues discussed the pros and cons of burnout instruments and concepts [2]. In addition to the well-established MBI, the authors recommended the Copenhagen Burnout Inventory (CBI, [63]), the Work-Related Behaviour and Experience Patterns (AVEM, [64]), the Oldenburg Burnout Inventory (OLBI, [65]) as alternatives to the MBI, depending on the specific aim of the study. Further studies should also analyse self-efficacy as a mediating variable and the effects on burnout resilience using other burnout measures or considering the single scales emotional exhaustion, cynicism and academic efficacy.

## Conclusions

Our results indicate the association of burnout resilience, on the one hand, and certain socio-demographic variables and variables related to medical school on the other. In addition, our results suggest the suppressor effect of self-efficacy in the relationship between prior vocational training in the medical field and burnout resilience. Medical students are at increased risk of burnout early in their studies. In response, international research stresses the need for student health management that teaches and promotes resilience, self-care, and empathy, in addition to medical knowledge and skills. It is of significant importance to foster and cultivate healthy, competent, empathetic students able to excel as doctors in the future [16]. Measures to promote resilience in healthcare students should extend beyond educating them about stressors and resilience factors. According to a recent Cochrane review [7], various interventions can be employed, and experiential exercises, including maintaining a self-care and well-being diary, could be included in a well-being portfolio [66].

Jerusalem and Schwarzer [36] have identified three focal points for curricular interventions: motivated learning, competent social behaviour, and proactive action. To promote academic, social, and general self-efficacy, individualised motivational strategies should be employed, the classroom climate should be improved, and competencies such as problem-solving skills and learning strategies should be acquired. Further studies could investigate the transferability of that intervention-based approach to medical students. Along similar lines, Van Dinther et al. [67] have demonstrated the effectiveness of interventions aimed at promoting students’ self-efficacy, particularly those based on social cognitive theory.

Because self-efficacy can be strengthened by peer support [68, 69], it may be beneficial to make the future study model more flexible and adaptable to students’ needs and thereby enable personalised teaching. The new admission regulations in Germany also allow for a more flexible design that integrates family and career into the degree program [70]. Further research is required to explore the link between professional training and the risk of burnout in medical studies in greater detail and to categorise it more effectively. In particular, the group of health professionals who will later work in a demanding environment involving direct or indirect contact with patients should receive training in maintaining their own mental health at an early stage. Beyond that, future intervention-based approaches should prioritise peer tutoring and personalised learning to educate and protect such a vulnerable group.

Overall, our study’s results provide important insights into the psychological mechanisms that modulate the relationship between vocational training, self-efficacy, and burnout resilience among medical students. The results highlight the importance of self-efficacy as a mediating variable and suggest that promoting it may be an effective strategy for improving burnout resilience among medical students.

## Data Availability

There are legal restrictions on the sharing of this de-identified dataset. The authors of the study received permission from the Medical Faculty of Tuebingen to collect the data only if they were not made publicly available without individual permission for specific questions (i.e. on request) due to the confidential nature of the data. Therefore, data are only available from the corresponding author: Dr. Rebecca Erschens or from Prof. Dr. med. Stephan Zipfel, Head of the Department for Psychosomatic Medicine and Psychotherapy, Medical University Hospital Tuebingen, Osianderstr. 5, 72076 Tuebingen/Germany.
